# The *DEP1* Mutation Improves Stem Lodging Resistance and Biomass Saccharification by Affecting Cell Wall Biosynthesis in Rice

**DOI:** 10.1186/s12284-024-00712-0

**Published:** 2024-05-15

**Authors:** Ye Wang, Meihan Wang, Xia Yan, Kaixuan Chen, Fuhao Tian, Xiao Yang, Liyu Cao, Nan Ruan, Zhengjun Dang, Xuelin Yin, Yuwei Huang, Fengcheng Li, Quan Xu

**Affiliations:** Key Laboratory of Crop Physiology, Ecology, Genetics and Breeding, Ministry of Agriculture, Shenyang Agricultural University, Shenyang, China

**Keywords:** Rice, Cell wall, *DEP1*, Lodging Resistance, Biomass Saccharification

## Abstract

**Background:**

Plant cell walls have evolved precise plasticity in response to environmental stimuli. The plant heterotrimeric G protein complexes could sense and transmit extracellular signals to intracellular signaling systems, and activate a series of downstream responses. *dep1* (*Dense and Erect Panicles 1*), the gain-of-function mutation of *DEP1* encoding a G protein γ subunit, confers rice multiple improved agronomic traits. However, the effects of *DEP1* on cell wall biosynthesis and wall-related agronomic traits remain largely unknown.

**Results:**

In this study, we showed that the *DEP1* mutation affects cell wall biosynthesis, leading to improved lodging resistance and biomass saccharification. The *DEP1* is ubiquitously expressed with a relatively higher expression level in tissues rich in cell walls. The CRISPR/Cas9 editing mutants of *DEP1* (*dep1-cs*) displayed a significant enhancement in stem mechanical properties relative to the wild-type, leading to a substantial improvement in lodging resistance. Cell wall analyses showed that the *DEP1* mutation increased the contents of cellulose, hemicelluloses, and pectin, and reduced lignin content and cellulose crystallinity (CrI). Additionally, the *dep1-cs* seedlings exhibited higher sensitivity to cellulose biosynthesis inhibitors, 2,6-Dichlorobenzonitrile (DCB) and isoxaben, compared with the wild-type, confirming the role of *DEP1* in cellulose deposition. Moreover, the *DEP1* mutation-mediated alterations of cell walls lead to increased enzymatic saccharification of biomass after the alkali pretreatment. Furthermore, the comparative transcriptome analysis revealed that the *DEP1* mutation substantially altered expression of genes involved in carbohydrate metabolism, and cell wall biosynthesis.

**Conclusions:**

Our findings revealed the roles of *DEP1* in cell wall biosynthesis, lodging resistance, and biomass saccharification in rice and suggested genetic modification of *DEP1* as a potential strategy to develop energy rice varieties with high lodging resistance.

**Supplementary Information:**

The online version contains supplementary material available at 10.1186/s12284-024-00712-0.

## Background

G protein (Guanine-nucleotide-binding protein) complex is a signal transduction molecule, consisting of Gα, Gβ and Gγ subunits, which widely exists on the plasma membrane of eukaryotic cells and plays important functions in various biological activities (Pandey 2019). Although the G protein signaling pathways in mammals are one of the best-understood pathways in the world, the underlying mechanisms related to G protein signaling in plants, especially in cell wall biosynthesis, have not been studied in depth. *DEP1* (*Dense and Erect Panicles 1*) encodes a Gγ subunit in rice, and its gain-of-function mutation, *dep1*, plays a critical role in super rice breeding in Northern China (Huang et al. 2009; Botella 2012; Xu et al. 2016). The disruption of *DEP1* has multiple positive effects on rice growth and development, including improvements in nitrogen utilization efficiency, panicle development, stress resistance, and lodging resistance (Huang et al. 2009; Sun et al. 2014; Xu et al. 2016; Zhao et al. 2016; Li et al. 2019; Cui et al. 2020). However, the mechanism underlying the positive effects of *dep1* on those traits remains largely unknown.

Plant cells are surrounded by cell walls that provide mechanical strength for plant upright growth, determine cell shape, and protect plant cells against various environmental stresses (Zhang et al. 2021). The plant cell wall is mainly composed of polysaccharides, lignin, as well as minor wall proteins. Cellulose, a major wall polysaccharide, constitutes the load-bearing skeleton of cell walls and provides flexibility and mechanical strength for plants (Somerville et al., 2004). Cellulose is biosynthesized by the cellulase synthase (CESA) complex at the plasma membrane (Lampugnani et al. 2019). Hemicellulose is the most abundant amorphous polysaccharide in plant cell walls and plays an important role in cell wall formation by interacting with cellulose and lignin (Hatfield et al. 2017). Xylans are the principal hemicellulosic polysaccharides, which consist of a linear β-(1,4)-linked xylosyl residue chain substituted with diverse glycosyl residues (Zhong et al. 2019). Lignin, an amorphous polymer composed of phenylpropane units connected by carbon-carbon bonds and ether bonds, is primarily deposited in the secondary cell wall and provides plant stiffness (Vanholme et al. 2019). The synthesis and polymerization of lignin are required for over 90 genes derived from 10 super-families (Raes et al. 2003; Yoon et al. 2015).

Stem lodging resistance is an important and comprehensive agronomic trait that directly determines crop grain yield and quality. Stem lodging resistance is closely associated with breaking force of basal stem internode, fresh weight, and plant height (Li et al. 2015). The Green Revolution program aims to improve crop lodging resistance by breeding semi-dwarf varieties with reducing plant height. However, dwarfing breeding limits the further increase of crop grain yield. Hence, breeders have made efforts to breed rice varieties with high lodging resistance by improving mechanical strength without compromising on plant height. Since cell wall composition and structure fundamentally determine plant mechanical strength as well as biomass enzymatic saccharification, the genetic modification of cell walls is suggested as a potentially promising strategy to simultaneously improve crop lodging resistance and biomass saccharification.

Few studies have been conducted to investigate the connection between G protein and cell wall biosynthesis in Arabidopsis. Transcriptome analysis showed that differentially expressed genes in Gγ and Gβ mutants are highly enriched in cell wall biosynthesis and modification processes (Delgado-Cerezo et al. 2012). Klopffleisch et al. (2011) demonstrated that cell wall xylose metabolism-associated enzymes could interact with G protein detected by the yeast two-hybrid technology. Mutations in Gγ and Gβ lead to decreased xylose content in Arabidopsis (Klopffleisch et al. 2011; Delgado-Cerezo et al. 2012), whereas knockout of both Gɑ and Gβ results in an increase in the xylose proportion in cell walls (Klopffleisch et al. 2011). In addition, a 7TM (seven-transmembrane domain) protein, annotated as a putative GPCR (G protein-coupled receptor) response for regulation of G protein signaling, was characterized to be required for cellulose biosynthesis in Arabidopsis, particularly during cell wall stress (McFarlane et al. 2021). However, the roles of G protein signaling in cell wall production in grass remains elusive.

Although much progress has been made in the study of G protein signaling in mammals, the underlying mechanisms associated with G protein γ subunits in plants, especially in cell wall biosynthesis, have yet to be explored. In this study, we investigated the effects of DEP1 on cell wall biosynthesis, lodging resistance, and biomass saccharification in rice by characterizing *DEP1* transgenic plants generated by the CRISPR/Cas9 approach. Our findings reveal that the *DEP1* mutation alters cell wall composition and structure in rice, leading to large improvements in stem lodging resistance and biomass enzymatic saccharification. This study also suggests *DEP1* as an elite gene resource for improving cell wall-relevant agronomic traits in rice.

## Methods

### Generation of Transgenic Rice and Growth Conditions

The *DEP1* gene editing transgenic lines (*dep1-cs*) were developed by using the CRISPR/Cas9 approach, single-guide RNA for generation of *dep1-cs1/2* (GCGCGAGATCACGTTCCTCA) and *dep1-cs3/4* (GGGGGCTAGAGCAGTTGCAC) lines were designed with the web-based tool CRISPR-PLANT (http://omap.org/crispr/CRISPRsearch.html) and was respectively ligated to the pRGEB32 vector. The constructed plasmids were transferred into wild-type rice (Sasanishiki) using the *Agrobacterium*-mediated transformation method. Positive transgenic plants obtained from CRISPR/Cas9 editing were identified by examination of hygromycin and Cas9 genes through PCR technology. Then, the different variations of positive transgenic lines were sequenced. The homozygous transgenic lines of T2 generation were used for further analysis. All rice plants used in this study were grown in a paddy field of Shenyang Agricultural University (Shenyang, China) in natural growing seasons.

### Gene Expression Analysis by Using the Real-Time Quantitative PCR (Q-PCR)

Total RNA extraction from fresh rice tissues and cDNA preparation were performed as described previously (Li et al. 2015). The primers used in this study for Q-PCR analysis are listed in Table S1.

### Determinations of Agronomic Traits and Stem Properties

The plant height, grain number per panicle, panicle length, tiller number, setting rate, 1000-grain weight, grain yield, and dry biomass were determined at the mature stage. Dry biomass, including stem, leaves, and leaf sheath, was weighed after the samples were dried to constant weight in an oven at 55℃. The stem properties, including lodging index, breaking force, puncture force, extension force, stem wall thickness, and diameter of stem internode, were examined 25 days after flowering. The mechanical characteristics of the stem were detected using a digital force/length tester (RH-K300). The stem lodging index was calculated as previously described (Miao et al. 2023).

### Analyses of Cell Wall Composition and Cellulose Crystallinity Index (CrI)

The rice stems were collected 25 days after flowering. The cell wall fractionation and quantification were performed as previously described (Dang et al. 2023) with minor modifications. In brief, the dry rice sample was ground into powder through a 60-mesh screen (0.25 mm × 0.25 mm) and then, respectively, removed soluble sugars, lipids, and starch by potassium phosphate buffer (pH 7.0), chloroform-methanol (1:1, v/v), and Dimethyl sulfoxide (DMSO) - water (9:1, v/v) step by step. After centrifugation at 3000 *g* for 5 min, the pellet was then washed twice with 75% (v/v) ethanol, and the residual pellet was dried to constant weight in an oven at 55℃ and was referred to as the alcohol insoluble residue (AIR). The pectin was then extracted from the AIR with the solution of ammonium oxalate (0.5% w/v) in a boiling water bath for 1 h. Following centrifugation, the hemicellulose was further extracted from the pellet with 4 N KOH (containing 1% NaBH_4_) for 1 h. After washed to be neutral with distilled water, the pellet, defined as crude cellulose, was dissolved in 72% H_2_SO_4_ (w/v). The hexose and pentose were determined as previously described (Li et al. 2013). The lignin content was determined as previously described (Li et al. 2014). The sugar composition of AIR was examined using GC-MS (7890 A-240; Agilent Technologies) as previously reported (Tang et al. 2022). All experiments were carried out in biological triplicate.

For the cellulose CrI assay, the crude cellulose obtained from cell wall fraction as described above was dried to constant weight and was used to examine cellulose CrI with the X-ray diffraction (Rigaku-D/MAX instrument, Ultima III; Japan) method as reported previously (Li et al. 2015).

### Immunochemical Analysis of Cell Wall Sugars

The immunochemical analyses of hemicellulosic xylans and pectin polysaccharides were carried out with antibodies LM10, LM11, and JIM7 as previously reported by Li et al. (2018)

### Analyses of Biomass Saccharification

The biomass enzymatic saccharification was quantified by calculating sugars released from mix-cellulases hydrolysis of biomass pretreated with 1% NaOH or 1% H_2_SO_4_, as previously described (Huang et al. 2012).

For observation of chemical-treated biomass residues in vitro, the biomass powder sample was treated with 1% NaOH for 2 h at 200 r/min in a shaking table at 50℃. After centrifugation at 3000 *g* for 5 min, the residual pellet was washed to be neutral with distilled water and dried to constant weight. The surface morphology of the dried sample was observed by scanning electron microscope observation (TM1000, Hitachi Ltd, Tokyo, Japan). All observations were conducted with three biological replicates, and each was observed three to five times, and a representative image was used in this study.

### Cellulose Biosynthesis Inhibitor Treatments

The germinated rice seeds were grown under normal hydroponic culture. After four days, half of the seedlings of wild-type and *dep1-cs* were treated with 500 nM isoxaben or 1500 nM 2,6-Dichlorobenzonitrile (DCB) (both of those were dissolved in methanol) for four days, and the remaining seedlings were maintained in hydroponic culture supplemented with 0.03% methanol as a control. The length of roots of wild-type and *dep1-cs* plants was calculated to reflect the sensitivity of the *DEP1* mutation to cellulose biosynthesis inhibitors.

### RNA Sequencing Analysis

Total RNA was extracted from the fresh tissues of the second stem internode of wild-type and *dep1-cs* plants at the booting stage. RNA quality check, the next-generation sequencing, and the subsequent differentially expressed gene (DEG) analysis and Kyoto Encyclopedia of Genes and Genomes (KEGG) enrichment analysis were carried out exactly as previously described by Ruan et al. (2022).

### Statistical Analysis

Data were presented as the mean ± SDs and numbers (*n*) of biological replicates were described in figure legends. Significance levels were tested using Student’s *t*-test and one-way ANOVA followed by a *post-hoc* test in SPSS Statistics software v.19.0. *P* values are indicated or labeled with different letters.

## Results

### *DEP1* is Highly Expressed in the Tissues Undergoing Cell Wall Biosynthesis

**Fig. 1:**
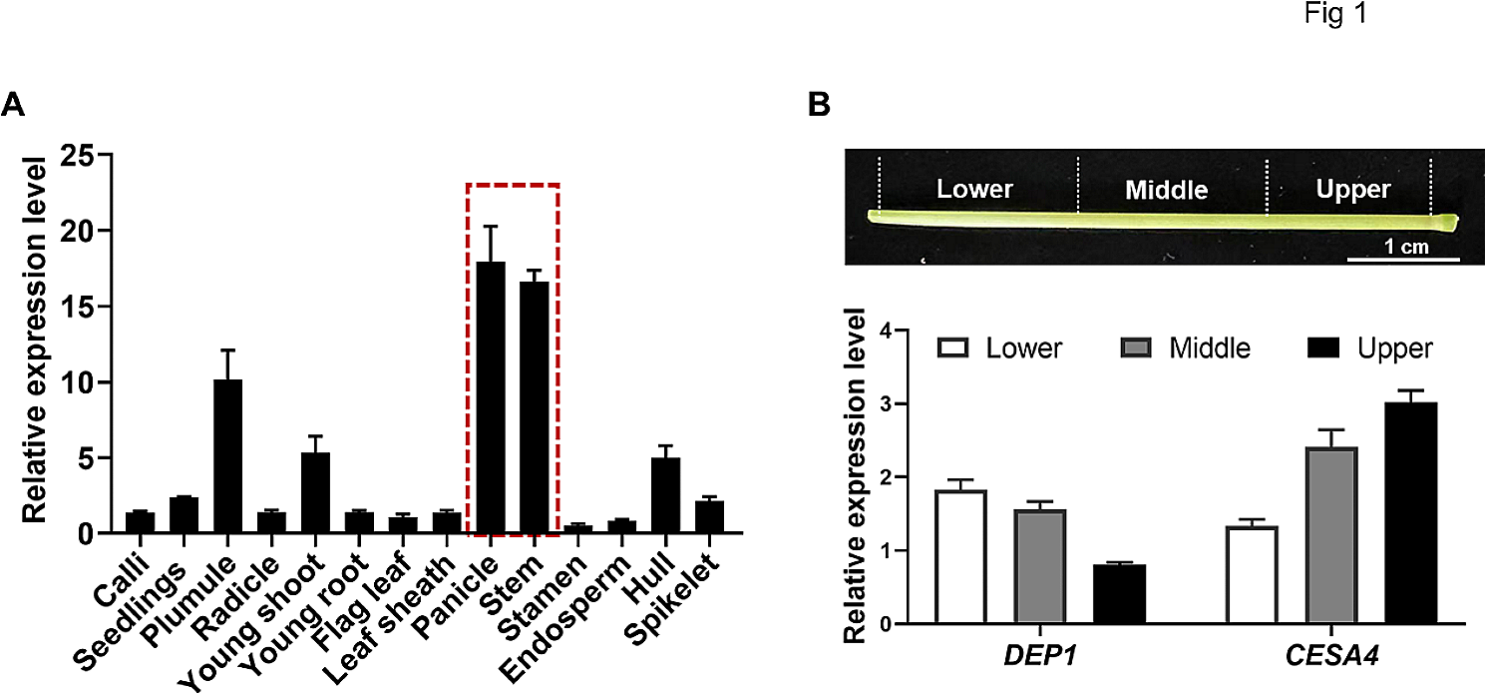
Expression pattern analysis of *DEP1* in rice. **A** The expression pattern of *DEP1* in various tissues examined by Q-PCR. **B** Quantification of expression levels of *DEP1* and *CESA4* genes in three sections (lower, middle, upper) of the second internode at the heading stage

Previous studies demonstrated the important role of *DEP1* in rice grain yield and nitrogen utilization (Huang et al. 2009; Sun et al. 2014). To further investigate whether *DEP1* is involved in cell wall biosynthesis, we examined the expression pattern of *DEP1* in various tissues and organs during rice growth. The *DEP1* showed the highest expression during the panicle development process (Fig. 1A), which corresponds with its well-documented role in panicle development (Huang et al. 2009). Notably, the *DEP1* gene is also highly expressed in the stem, which is enriched in cell walls. By contrast, the expression level of *DEP1* was lower in leaves, calli, radicle, young root, and endosperm tissues, which contain lower levels of cell walls (Fig. 1A). These data implied that the *DEP1* is closely associated with cell wall biosynthesis in rice. To investigate the potential function of *DEP1* in cell wall biosynthesis, we examined the expression pattern of *DEP1* during rice stem internode development, which has been established as a model for studying cell wall synthesis (Ruan et al. 2022). The stem internode of wild-type at the heading stage was divided into three sections (lower, middle, and upper, counting from bottom to the top), which represents the transition from the primary wall to the secondary wall (Zhang et al. 2017). The lower section contains cells undergoing elongation and the formation of primary and secondary cell walls, the middle section is in the state of rapid biosynthesis of secondary walls, and the cells in the upper section cease elongating and complete the secondary wall thicken (Zhang et al. 2017). Q-PCR analysis revealed that the *DEP1* is highly expressed in all three sections with a relatively higher expression level in the lower and middle sections (Fig. 1B). By comparison, the *CESA4* gene required for the secondary wall cellulose biosynthesis exhibited the highest expression level in the third section undergoing the secondary cell wall deposition (Fig. 1B). Collectively, these results suggest that *DEP1* probably contributes to cell wall biosynthesis.

### Generation of *DEP1* Knockout Mutants by the CRISPR/Cas9 System

**Fig. 2:**
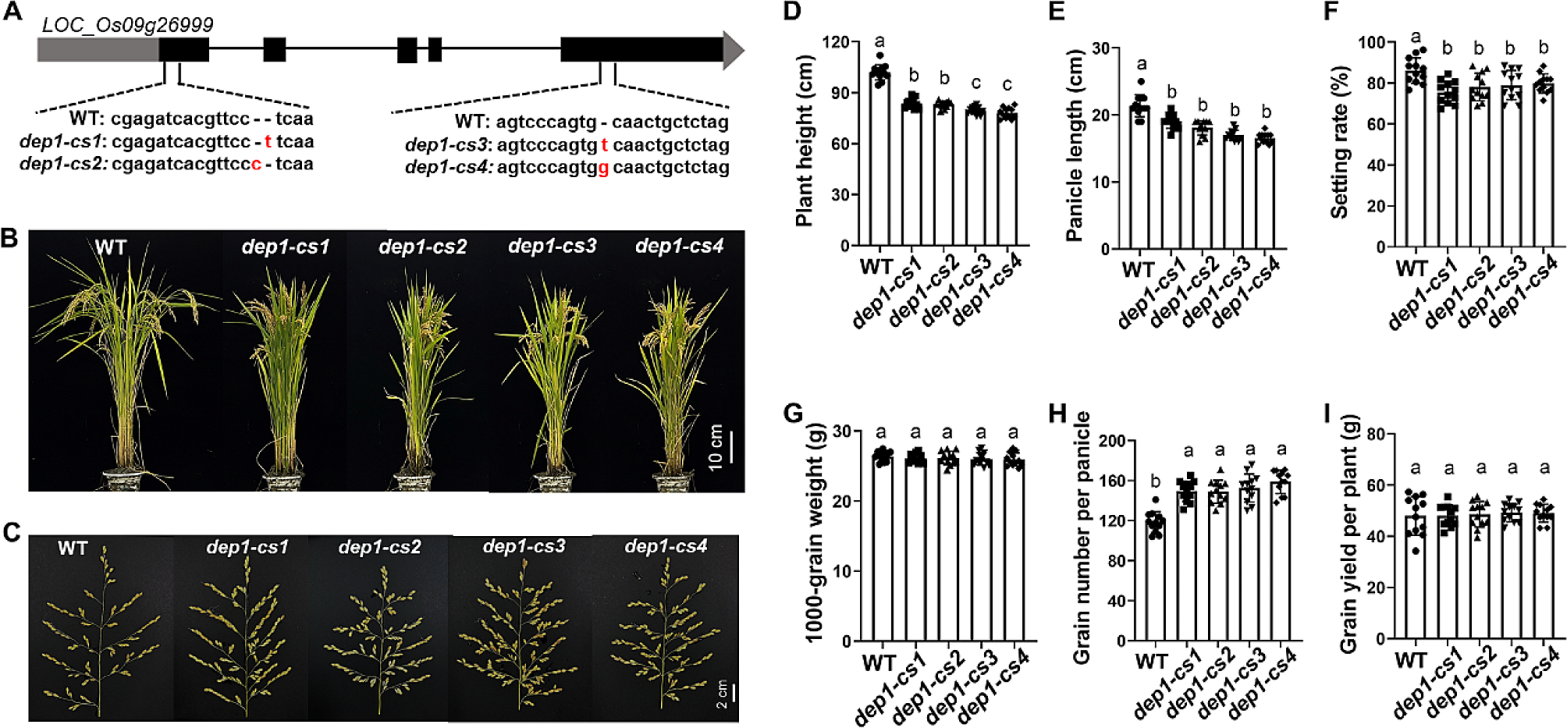
Phenotypes and agronomic traits of wild-type (WT) and *DEP1* transgenic lines (*dep1-cs*) generated by the CRISPR/Cas9 system. **A** Generation of *dep1-cs* mutants. **B, C** Phenotypes **(B)** and panicle performances **(C)** of WT and *dep1-cs* plants. **D-I** Agronomic traits of WT and *dep1-cs* plants, including plant height **(D)**, panicle length **(E)**, setting rate **(F)**, 1000-grain weight **(G)**, grain number per panicle **(H)**, and grain yield per plant **(I)**. Values are means, error bars are SD; *n* = 12 biological replicates; Significant differences are indicated by different letters (*P* < 0.05)

To genetically investigate the biological function of *DEP1*, four independent gene editing transgenic lines, *dep1-cs1*, *dep1-cs2*, *dep1-cs3*, and *dep1-cs4*, were generated on a *japonica* variety, Sasanishiki (wild-type) background by using the CRISPR/Cas9 system. The *dep1-cs1* and *dep1-cs2*, had one base pair insertion in the first exon, leading to a premature stop codon and causing a very short truncated protein; while the *dep1-cs3* and *dep1-cs4* showed one base pair insertion in the cysteine-rich region of the fifth exon at the C-terminus, resulting in the loss of the cysteine‐rich domain (Fig. 2A; Fig. S1). Compared to wild-type plants, all *dep1-cs* lines showed a significant reduction in plant height and panicle length (Fig. 2B-E), consistent with previous reports (Li et al. 2019). The *dep1-cs3* and *dep1-cs4* plants, containing a truncated DEP1 protein without cysteine-rich region, displayed a shorter plant height than *dep1-cs1* and *dep1-cs2* lines containing completely knockout *DEP1* (Fig. 2B, D). While panicle length and setting rate were significantly decreased without difference in 1000-grain weight (Fig. 2E-G), the grain number per panicle was significantly higher in *dep1-cs* lines relative to wild-type plants (Fig. 2H), resulting in a similar grain yield per plant (Fig. 2I). Additionally, tiller number was increased in the *dep1-cs* lines compared to wild-type plants (Fig. S2A), whereas no substantial differences observed in biomass production (Fig. S2B).

### The *DEP1* Mutation Increases Stem Mechanical Strength for High Lodging Resistance

**Fig. 3:**
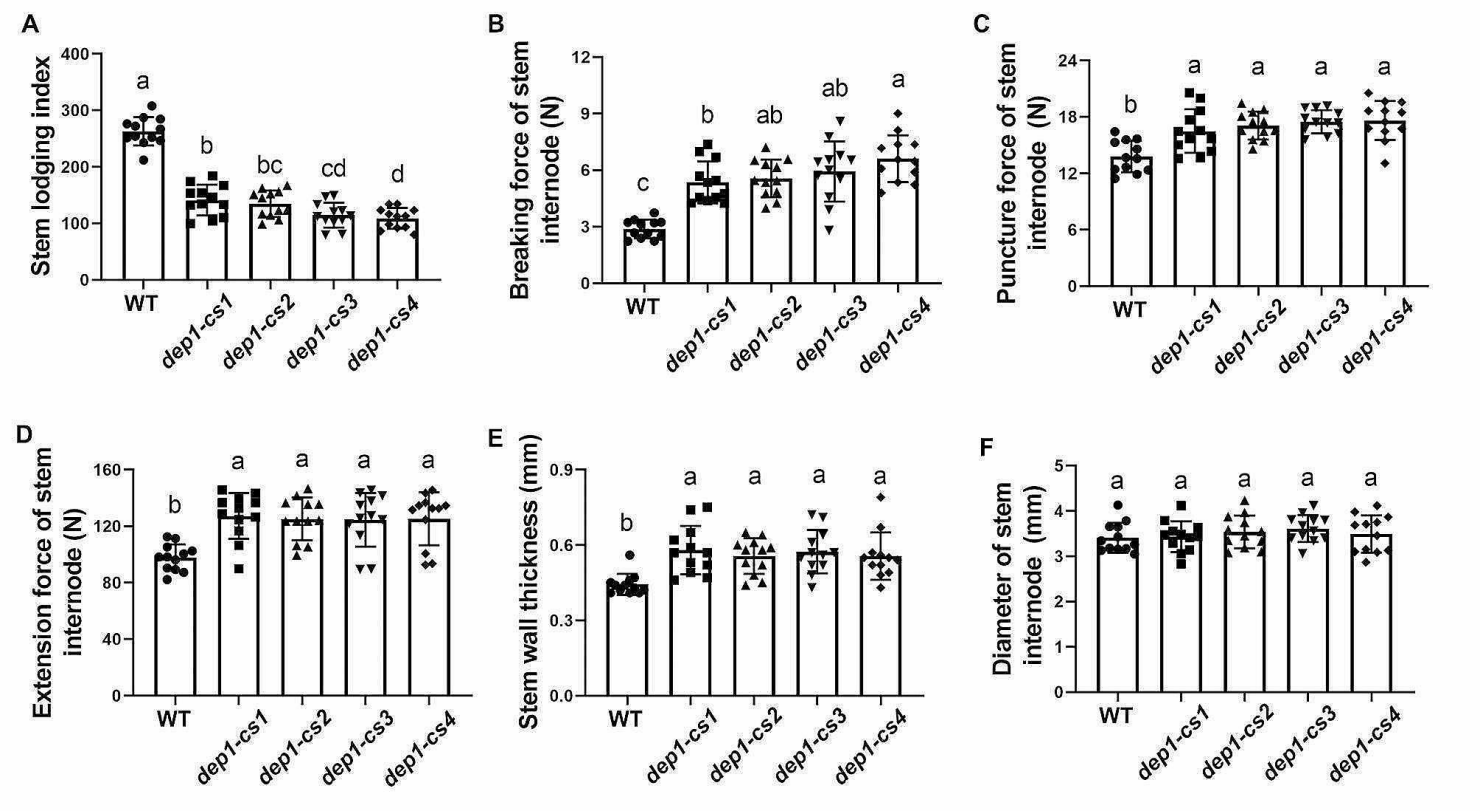
Measurement of lodging index and mechanical strength associated properties of wild-type (WT) and *dep1-cs* plants. **A** Stem lodging index. **B** Breaking force, **(C)** puncture force, and **(D)** extension force of stem internode. **E** Stem wall thickness. **F** Diameter of stem internode. Values are means, error bars are SD; *n* = 12 biological replicates; Significant differences are indicated by different letters (*P* < 0.05)

Notably, compared to the wild-type, the *dep1-cs* mutants showed a substantial decrease in the lodging resistance index, a parameter negatively correlated to plant lodging resistance ability (Fig. 3A). As plant lodging resistance is negatively determined by plant height and fresh weight and is positively determined by basal stem breaking force (Liu et al. 2018), we further compared the mechanical properties of *dep1-cs* lines with wild-type plants. As a result, the *dep1-cs* lines showed a significant increase in multiple mechanical properties of stem internode, including breaking force (Fig. 3B), puncture force (Fig. 3C), and extension force (Fig. 3D). Additionally, the stem wall thickness of basal internodes in *dep1-cs* lines was significantly increased compared to that in the wild-type plants, whereas no obvious alteration was observed in the diameter of basal stem internode (Fig. 3E, F). Hence, the substantially increased mechanical strength caused by the *DEP1* mutation leads to a significant improvement in lodging resistance in *dep1-cs* lines.

### DEP1 Affects Cell Wall Structure and the Content of Wall Polymers

**Fig. 4:**
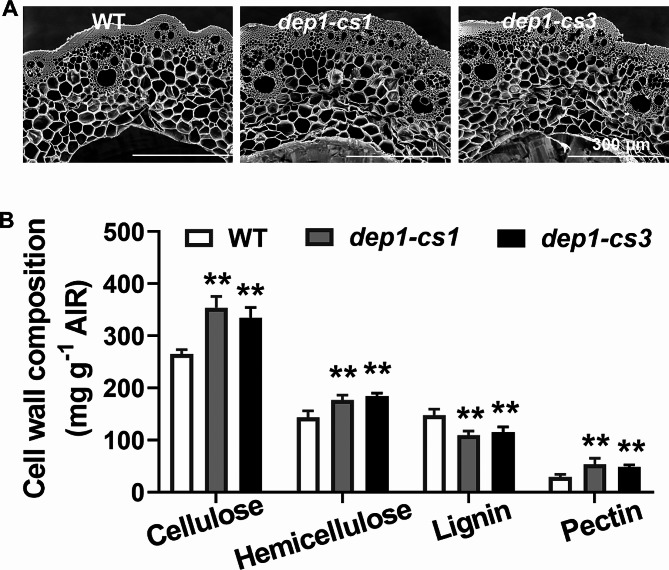
Morphology of xylem cell walls and determination of cell wall polymers of wild-type (WT) and *dep1-cs* plants. **A** Morphology of xylem cell walls of WT and *dep1-cs* plants. **B** Determination of cell wall polymers of WT and *dep1-cs* plants. **P < 0.01. Student’s *t*-test, values are means, error bars are SD, *n* = 3 biological replicates

The significant enhancement of stem lodging resistance and stem wall thickness by the *DEP1* mutation promotes us to investigate the effect of *DEP1* on cell wall composition and structure, which largely determines plant mechanical strength. The cross-sections of the second internodes (counting from the top) were harvested from wild-type and *dep1-cs* plants at the heading stage and were visualized by the SEM. Except for thicker stem walls, there was no significant change in the vascular structure of the stem in the *dep1-cs* lines relative to wild-type plants (Fig. 4A). Furthermore, we performed cell wall polymer extraction and analysis using the mature stem tissue harvested from the wild-type and *dep1-cs* plants. The cellulose, hemicellulose, and pectin content in the *dep1-cs* lines was significantly increased (30%, 26%, and 70%, respectively), while a 24% reduction was observed in the lignin level compared with the wild-type plants (Fig. 4B). Taken together, these data suggested that DEP1 is highly associated with the biosynthesis of cell wall polymers in rice.

### The *DEP1* Mutation Affects Sugar Composition and Cellulose Structure

**Fig. 5:**
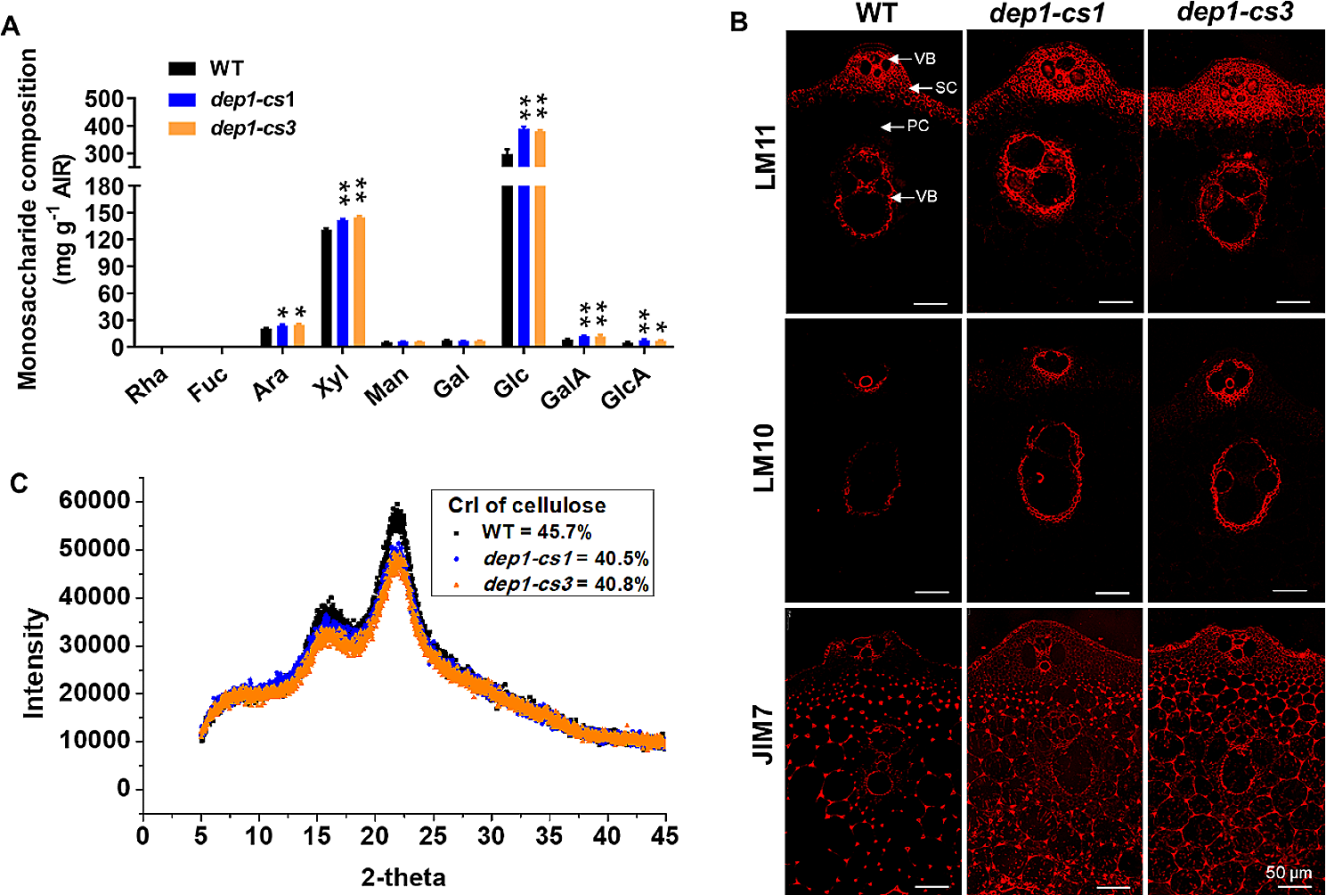
Sugar composition and cellulose structural feature of wild-type (WT) and *dep1-cs* plants. **A** Monosaccharide composition of cell wall carbohydrates for WT and *dep1-cs* plants. AIR, alcohol-insoluble residues. *P < 0.05, **P < 0.01. Student’s *t*-test, values are means, error bars are SD, *n* = 3 biological replicates. **B** Immunochemical staining of cross-sections of WT and *dep1-cs* plant stem internodes. Representative micrographs of equivalent sections of mature culm internodes immunolabeled with antibodies directed to xylan (LM11 and LM10) and homogalacturonan (JIM7). PC, parenchyma cells; SC, sclerenchyma cells; VB, vascular bundle cells. **C** Determination of lignocellulose CrI of mature stems using the X-ray diffraction method

Since the DEP1 plays a role in the content of cell wall polymers, we further investigated whether the *DEP1* mutation also affected sugar composition and cellulose features. The GC-MS technology was used to compare the monosaccharide composition of cell walls of mature stem tissues harvested from wild-type and *dep1-cs* plants. Compared to the wild-type plants, the *dep1-cs* lines showed a considerable increase in glucose, xylose, and arabinose, consistent with their increased content of cellulose and hemicellulose (Fig. 5A). In addition, the content of GlcA and GalA, two primarily monosaccharides of pectin, in the *dep1-cs* lines was also significantly increased (Fig. 5A). To confirm the alterations of sugar composition in *dep1-cs* lines, we performed immunochemical analysis of transverse sections of culm internodes labeled with antibodies LM11 and LM10 binding to xylan, and JIM7 directed to homogalacturonan. As a result, the fluorescent signals of all antibodies LM10, LM11, and JIM7 in the *dep1-cs* internodes were stronger than those in the wild-type plants (Fig. 5B), confirming the alterations of polysaccharides in the *dep1-cs* lines examined by the cell wall polymer fractionation and analysis. Moreover, we analyzed the CrI of cellulose extracted from mature stems of the wild-type and *dep1-cs* plants. The cellulose CrI of the *dep1-cs* plants was decreased by approximately 11% compared with that of the wild-type plants (Fig. 5C). Taken together, these results revealed an important role of *DEP1* in the biosynthesis of polysaccharides.

**Fig. 6:**
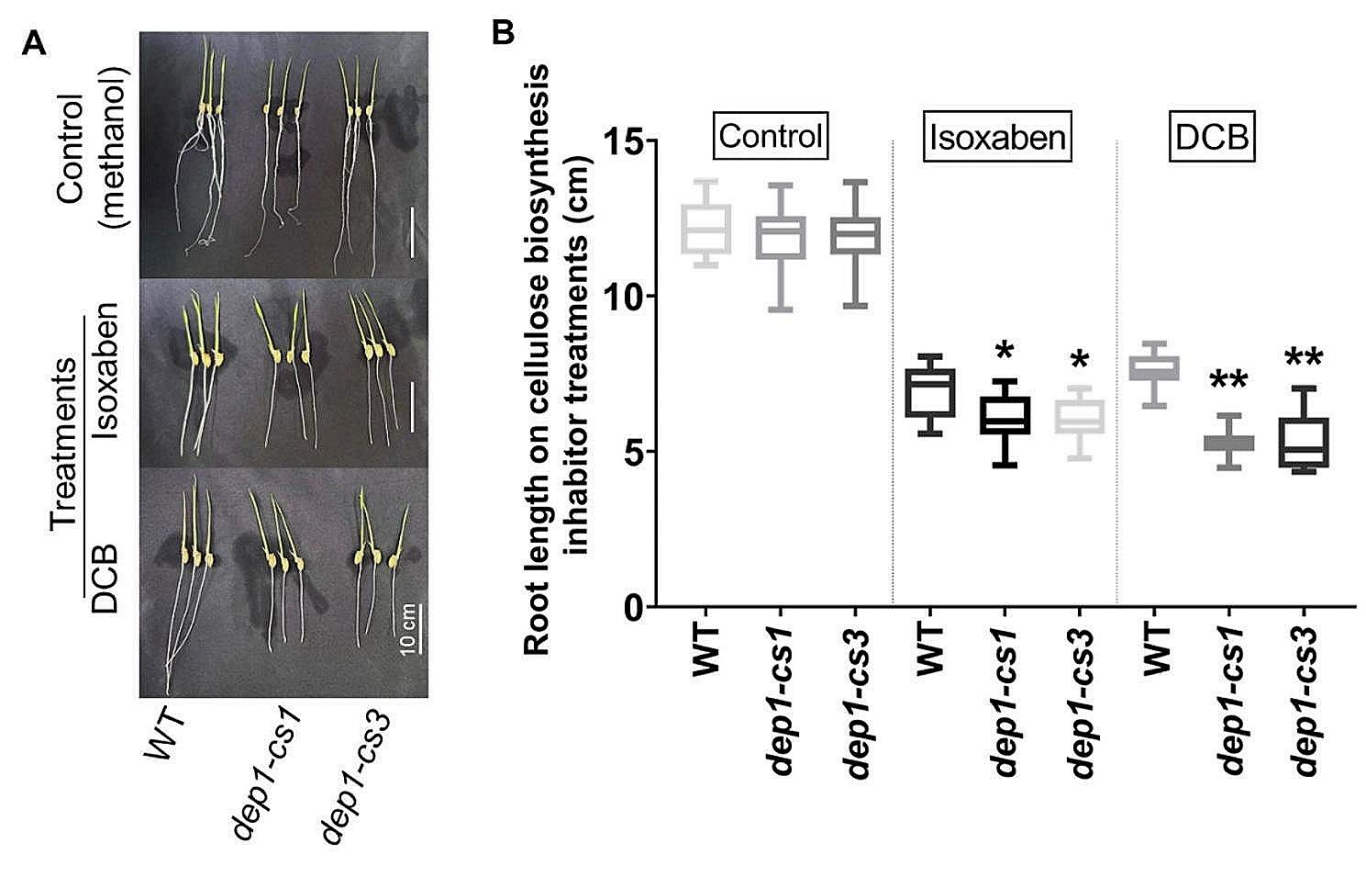
The *dep1-cs* plants display increased sensitivity to cellulose biosynthesis inhibitors. **A** Representative images of five-day-old rice seedlings grown under hydroponic culture supplemented with 500 nM isoxaben or 1500 nM 2,6-Dichlorobenzonitrile (DCB). **B** Quantification of root lengths of seedlings represented in (A). *n* = 3 biological replicates, each comprising 6 plants, *P < 0.05, **P < 0.01, Student’s *t*-test

To further confirm the association of DEP1 with cellulose biosynthesis, we treated the three-day-old *dep1-cs* and wild-type seedlings with isoxaben and 2,6-Dichlorobenzonitrile (DCB), two cellulose biosynthesis inhibitors, for six days. Under unstressed conditions, there was no significant alteration in the root length between wild-type and *dep1-cs* plants (Fig. 6). By contrast, the *dep1-cs* lines displayed an increased sensitivity to both isoxaben and DCB. In detail, compared to wild-type plants, the root length was reduced by up to approximately 14% and 31% in *dep1-cs* seedlings exposed to isoxaben and DCB, respectively (Fig. 6). These data suggest an important role of DEP1 in cellulose biosynthesis, particularly, during cell wall stress.

### The *DEP1* Mutation Leads to an Enhancement in Biomass Saccharification

**Fig. 7:**
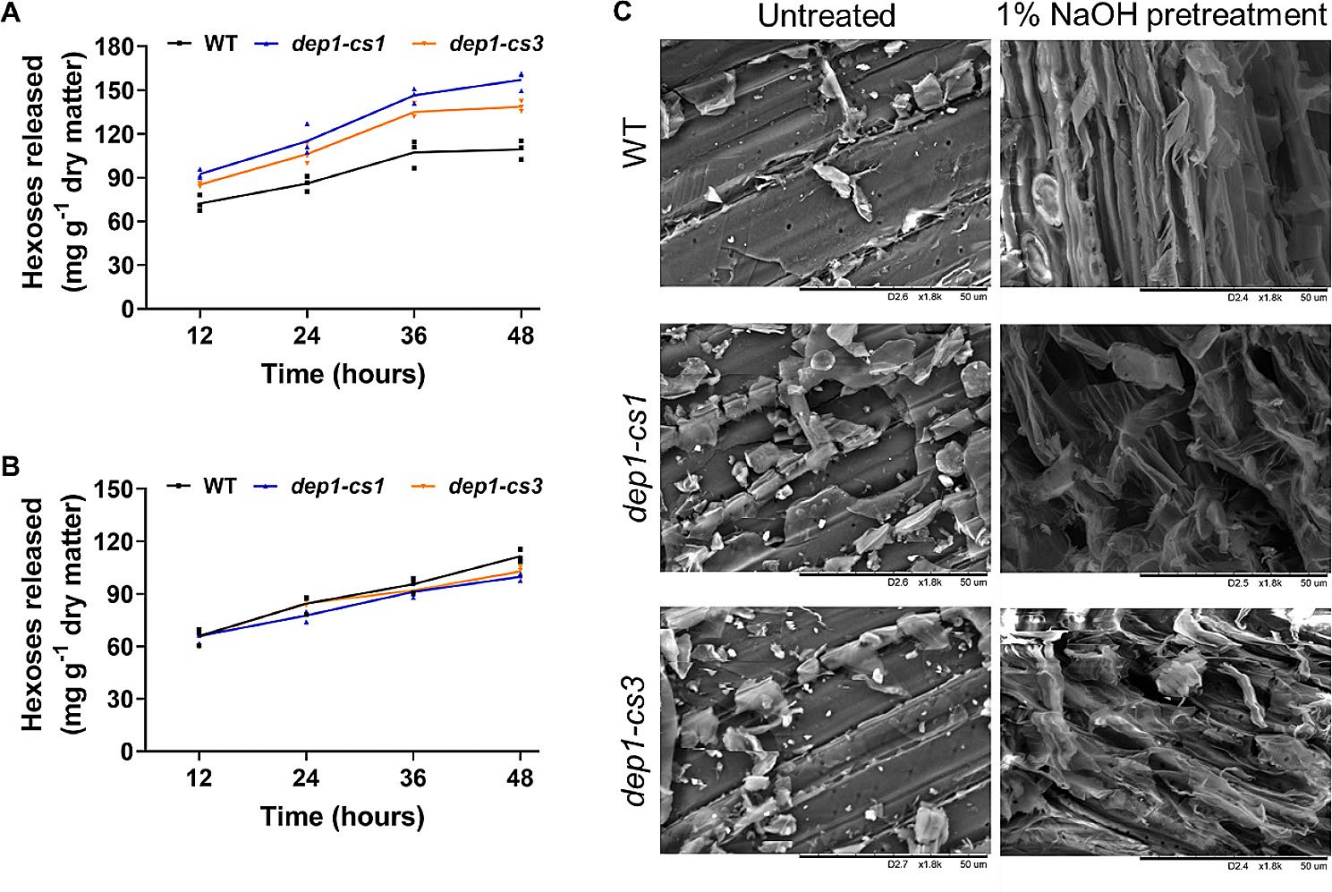
Biomass enzymatic saccharification of wild-type (WT) and *dep1-cs* plants. **A, B** Hexose yields released from enzymatic (mixed-cellulases) hydrolysis after pretreatment with 1% NaOH (**A**) or 1% H_2_SO_4_ (**B**). **C** Scanning electron microscopy (SEM) images of the surface of biomass after 1% NaOH pretreatment

The altered cell wall composition and reduced cellulose CrI promote us to investigate the biomass enzymatic saccharification of the *dep1-cs* mutants. We used two chemical reagents, alkali (1% NaOH) or acid (1% H_2_SO_4_), to pretreat the powders of the mature straw. We then compared the biomass saccharification by measuring hexoses released from mix-cellulases hydrolysis of pretreated mature straw. After NaOH pretreatment, both the *dep1-cs* lines displayed a significantly higher biomass enzymatic hydrolysis efficiency than the wild-type (Fig. 7A). By contrast, there was no obvious change in hexose yield released from enzymatic hydrolysis of biomass after H_2_SO_4_ pretreatment (Fig. 7B).

To explain the improved saccharification of the NaOH-pretreated biomass in the *dep1-cs* lines, we used SEM to scan the surface morphology of biomass residues under the control conditions or 1% NaOH treatment. There was no discernable difference in biomass residue surfaces without pretreatment between wild-type and *dep1-cs* plants (Fig. 7C). Notably, rougher surface of the NaOH-pretreated biomass residue in *dep1-cs* mutants was observed as compared with that in the wild-type (Fig. 7C). These data indicate that the *DEP1* mutation produces a rougher surface of biomass residue under the alkali treatment, which increases the enzyme accessibility to lignocellulose for higher biomass saccharification in *dep1-cs* mutants.

### The *DEP1* Mutation Results in Substantial Transcriptional Changes in Cell Wall Biosynthesis-Related Genes

**Fig. 8:**
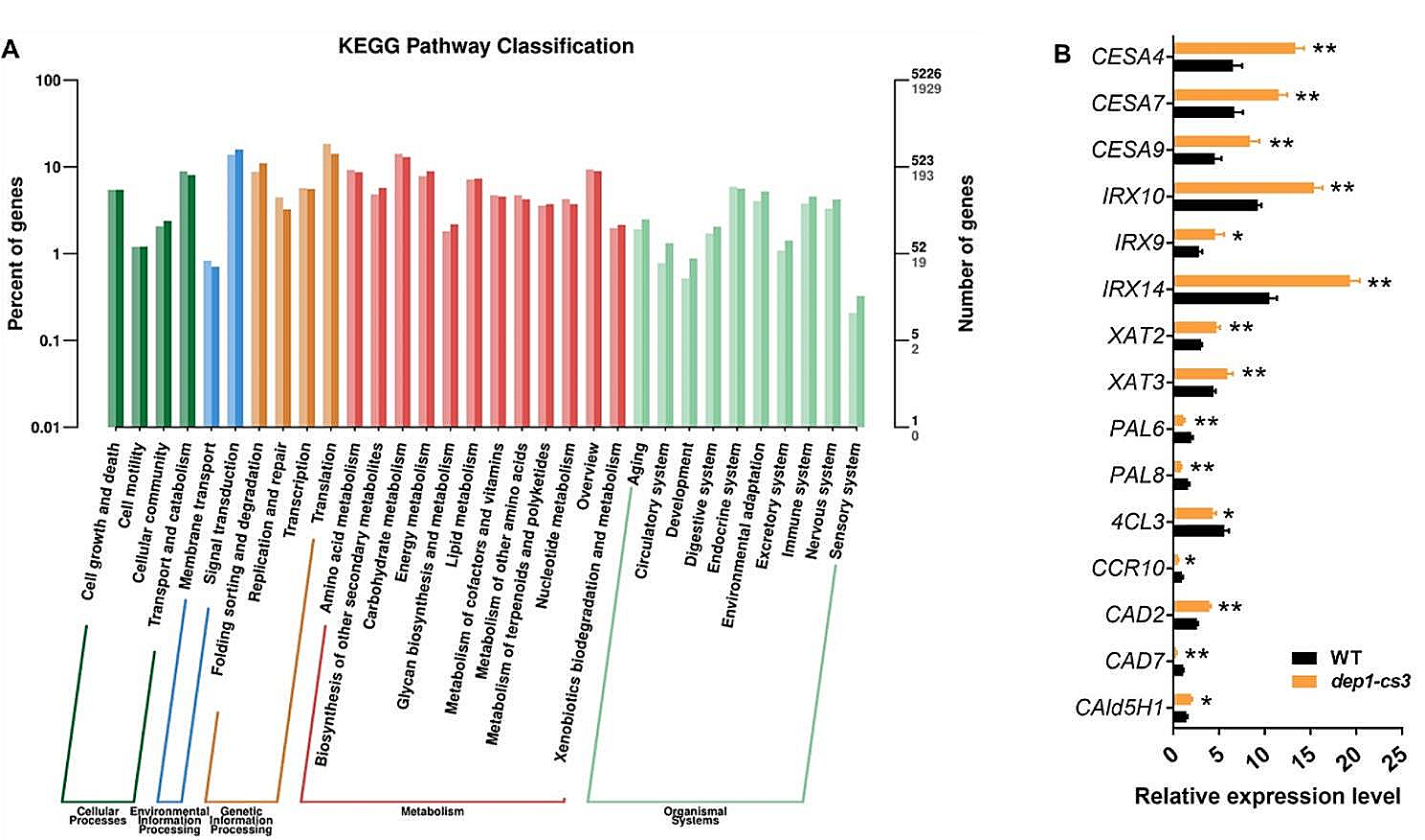
Transcriptome profile of stem internodes in wild-type (WT) and *dep1-cs* plants. **A** KEGG enrichment analysis was performed to identify the altered pathways of differentially expressed genes based on RNA-sequencing analysis. **B** The relative expression levels of genes involved in cell wall biosynthesis in WT and *dep1-cs* plants quantified by Q-PCR. *P < 0.05, **P < 0.01. Student’s *t*-test, values are means, error bars are SD, *n* = 3 biological replicates

The RNA-Seq technology was used to compare transcriptional profiling of the stems of wild-type and *dep1-cs3* plants at the heading stage. A total of 9955 DEGs (Differentially Expressed Genes) (*P* < 0.05, fold change ≥ 1.5) were found in the *dep1-cs3* mutant compared to the wild-type, of which 5057 genes were upregulated and 4898 genes were downregulated (Fig. S3), corresponding to the involvements of *DEP1* in many biological processes. Moreover, the Kyoto Encyclopedia of Genes and Genomes (KEGG) enrichment analysis revealed that the DEGs participate in up to 268 pathways, of which metabolisms of carbohydrates, amino acids, lipids, nucleotides, and vitamins were substantially affected (Fig. 8A). Among the carbohydrate metabolism-related DEGs, many genes have been recognized or annotated to be involved in cell wall polysaccharide formation. We further performed a Q-PCR analysis to examine the influences of the *DEP1* mutation on the expression of genes involved in cell wall polymer biosynthesis and modification. The expression levels of *CESA4/7/9* required for secondary wall cellulose biosynthesis in rice were significantly increased in the *dep1-cs3* mutant, consistent with its higher cellulose content compared to wild-type plants (Fig. 8B). With regard to hemicellulose biosynthesis, we examined the expression of genes contributing to the elongation of the xylan backbone (*IRX9*, *IRX10*, *IRX14*) (Chiniquy et al. 2013; Dang et al. 2023), and the addition of sidechain (*XAT2*, *XAT3*) (Anders et al. 2012). All these genes were upregulated in the mutant, consistent with the increased contents of hemicellulose, arabinose, and xylose in *dep1-cs* lines (Fig. 8B). Based on the reduced lignin level in the mutant, we further analyzed the transcriptional level of lignin metabolism-related genes. The *PAL6*, *PAL8*, *4CL3*, *CCR10*, and *CAD7* genes were downregulated in the mutant, whereas the *CAD2* and *CAld5H1* were upregulated, implying a regulatory role of *DEP1* in the biosynthesis of lignin monomers (Fig. 8B).

## Discussion

**Fig. 9:**
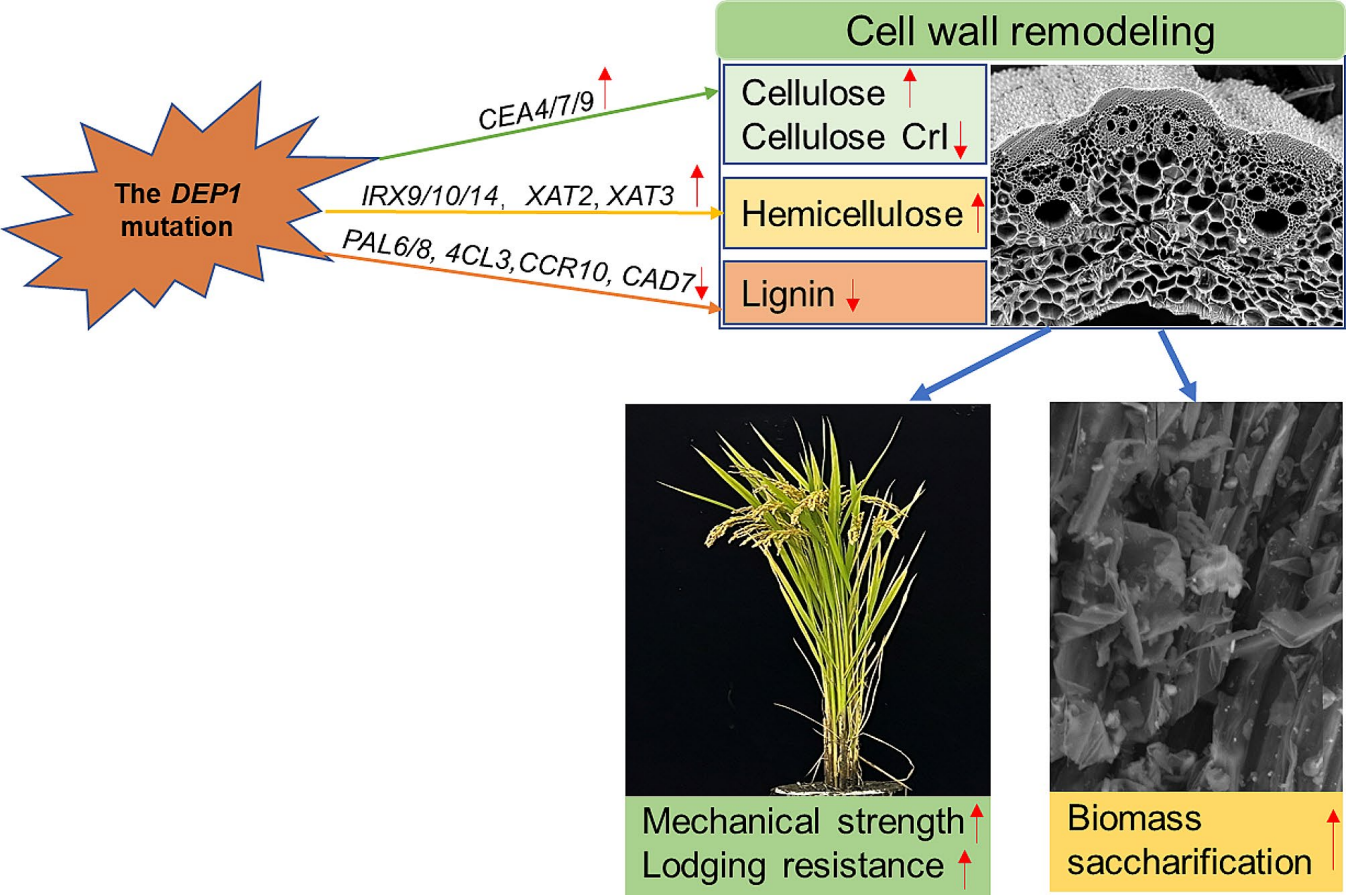
A hypothetic model to elucidate the *DEP1* mutation-mediated cell wall remodeling for improvements in stem lodging resistance and biomass saccharification

As a hub of signal reception and transfer, the G protein signaling is involved in the regulation of almost all biological processes in plants, including cell division, defense to stresses, switch of ion channels, perception of sugar signals, and various plant hormone signaling processes (Urano et al. 2013). However, the effect of G protein signaling on cell wall production, which fundamentally determines plant mechanical strength and biomass saccharification, is still unknown. In this work, we examined the roles of a G protein γ subunit, DEP1, in cell wall biosynthesis and cell wall-related traits in rice. We propose that the DEP1 mutation leads to cell wall remodeling by affecting expression levels of cell wall biosynthesis-related genes, which enhances biomass enzymatic saccharification and improves stem mechanical strength for high lodging resistance (Fig. 9).

### DEP1 Plays a Role in Cell Wall Remodeling in Rice

In this study, we demonstrated that DEP1 functions in cell wall remodeling. The *DEP1* gene is highly expressed in cell wall-rich tissues and the *DEP1* mutation leads to altered expression levels of genes involved in cell wall biosynthesis. Similarly, a previous report revealed that the mutation in Gβ or Gγ leads to similar transcriptomic alterations, which are enriched in cell wall biosynthesis-related genes (Delgado-Cerezo et al. 2012). These data suggest that *DEP1* is involved in cell wall production at the transcriptional level. Moreover, the *dep1-cs* lines showed a significant increase in sugar contents, including cellulose, hemicellulose, pectin, xylose, arabinose, and glucose, and a decrease in lignin, confirming the significant role of DEP1 in cell wall production. Consistently, the mutations in Arabidopsis G proteins result in altered contents of xylose and xylan (Klopffleisch et al. 2011; Delgado-Cerezo et al. 2012). Additionally, the *dep1-cs* lines exhibited more sensitivity to cellulose biosynthesis inhibitors, DCB, and isoxaben, relative to wild-type plants. A similar effect of G protein signaling on the regulation of cellulose synthesis was also observed in Arabidopsis (McFarlane et al. 2021). The mutation in the 7tm protein, interacting with the G protein complex, affects cellulose synthase complex (CSC) trafficking and thus impairs cellulose biosynthesis, particularly during cell wall stress (McFarlane et al. 2021). Furthermore, the *DEP1* mutation also results in a significantly reduced cellulose CrI, an important structural parameter of cellulose. Hence, our findings demonstrate that DEP1 is involved in the biosynthesis and modification of cell wall polymers in rice.

### The *DEP1* Mutation Enhances Stem Mechanical Strength for Improved Lodging Resistance

Although positive roles of *dep1* on rice growth performances in terms of panicle development, nitrogen utilization efficiency, and stress tolerance have been widely reported (Huang et al. 2009; Sun et al. 2014; Xu et al. 2016; Zhao et al. 2016; Li et al. 2019; Zhang et al. 2019; Cui et al. 2020), the underlying mechanisms connecting DEP1 with those traits remain largely unknown. In this study, we confirmed the negative role of DEP1 in stem lodging resistance by performing a genetic analysis. Notably, in addition to the reduction of plant height, the significantly increased mechanical strength caused by the *DEP1* mutation-mediated reinforcement of cell walls should be another essential contributor to improved stem lodging resistance in *dep1-cs* plants. As the cell wall functions as a determinant of mechanical strength, the substantial increase in wall polysaccharides of *dep1-cs* lines enhances the breaking force for high lodging resistance. Taken together, the *DEP1* mutation increases the contents of cell wall polymers, which reinforces the cell walls to enhance mechanical strength for a large improvement in stem lodging resistance.

### The *DEP1* Mutation Significantly Increases the Enzymatic Hydrolysis of Biomass After Alkali Pretreatment

It is well known that cell wall composition and structural feathers of wall polymers play essential roles in biomass saccharification. We found that the *DEP1* mutation leads to a significant increase in biomass enzymatic hydrolysis under alkali pretreatment. The increased biomass saccharification in *dep1-cs* lines was supported by their altered cell walls. First, the *DEP1* mutation substantially increases the contents of polysaccharides, especially cellulose, which provides more substrates for enzymatic hydrolysis reaction after pretreatment. Secondly, the reduction of cellulose CrI, a negative factor for biomass enzymatic hydrolysis, in *dep1-cs* mutants also contributes to the improvement of biomass saccharification by increasing cellulase accessibility to alkali-pretreated biomass.

Interestingly, the alkali pretreatment increased the yield of hexose from the *dep1-cs* biomass via enzymatic saccharification compared with that from the wild-type plants, whereas the sulfuric acid pretreatment did not. Inorganic alkali and acid treatments are two efficient pretreatment methods for biomass conversion, and they reduce the recalcitrance of lignocellulose, increasing the accessibility of cellulases with distinct mechanisms (Abraham et al. 2020). The acid treatment could dissociate the strong chemical bonds and partially release lignin monomers, hemicellulosic monosaccharides, and amorphous cellulose (du Pasquier et al. 2023). By comparison, alkali treatment could destroy cell wall polymers by eliminating crosslinked hemicellulose and lignin and decreasing cellulose CrI (Lee et al. 2015). As the *dep1-cs* plants contain a lower lignin content and a higher level of cellulose, the alkali treatment might further increase the proportion of amorphous cellulose and decrease cellulose CrI, resulting in significantly enhanced lignocellulose saccharification in mutants compared to wild-type plants. In contrast, the acid treatment might partially remove amorphous cellulose and lignin, reducing the advantage of lower cellulose CrI and lignin content for high biomass saccharification in *dep1-cs* plants, resulting in similar hexose yield from enzymatic hydrolysis of acid-treated biomass of *dep1-cs* compared to wild-type plants.

## Conclusions

Our findings show that the *DEP1* mutation improves both stem lodging resistance and biomass saccharification by remodeling cell wall deposition, including increased levels of cellulose, hemicellulose, and pectin, and reduced lignin content and cellulose CrI. This study provides a promising gene resource, *DEP1*, for genetic modification of cell walls to breed robust rice varieties with high straw biomass enzymatic saccharification.

### Electronic Supplementary Material

Below is the link to the electronic supplementary material.

Supplementary Material 1: Figure S1. Sequencing confirmation of the *DEP1* gene in WT and *dep1-cs* transgenic plants generated by the CRISPR/Cas9 approach. Figure S2. Agronomic traits of wild-type (WT) and *dep1-cs* plants. **A** Tiller number per plant. **B** Dry biomass per plant. Values are means, error bars are SD; *n* = 12 biological replicates; Significant differences are indicated by different letters (*P* < 0.05). Figure S3. The visualization of differentially expressed genes (DEG) between wild-type and *dep1-cs* plants was performed by volcano plot, with the magnitude of the fold-change shown along the X-axis and the statistical significance (-log10 of *q*-value) shown on the Y-axis. Table S1. Primers used in this study

## Data Availability

No datasets were generated or analysed during the current study.
