# Supplementary material for: The DEP1 Mutation Improves Stem Lodging Resistance and Biomass Saccharification by Affecting Cell Wall Biosynthesis in Rice
Source: Rice (N Y). 2024 May 15;17:35. doi: 10.1186/s12284-024-00712-0 (PMC11096150; doi:10.1186/s12284-024-00712-0)
Supplement: Supplementary file 1 — Supplementary Material 1: Figure S1. Sequencing confirmation of the DEP1 gene in WT and dep1-cs transgenic plants generated by the CRISPR/Cas9 approach. Figure S2. Agronomic traits of wild-type (WT) and dep1-cs plants. A Tiller number per plant. B Dry biomass per plant. Values are means, error bars are SD; n = 12 biological replicates; Significant differences are indicated by different letters (P < 0.05). Figure S3. The visualization of differentially expressed genes (DEG) between wild-type and dep1-cs plants was performed by volcano plot, with the magnitude of the fold-change shown along the X-axis and the statistical significance (-log10 of q-value) shown on the Y-axis. Table S1. Primers used in this study [file 12284_2024_712_MOESM1_ESM.pdf]

Supplemental figures and tables

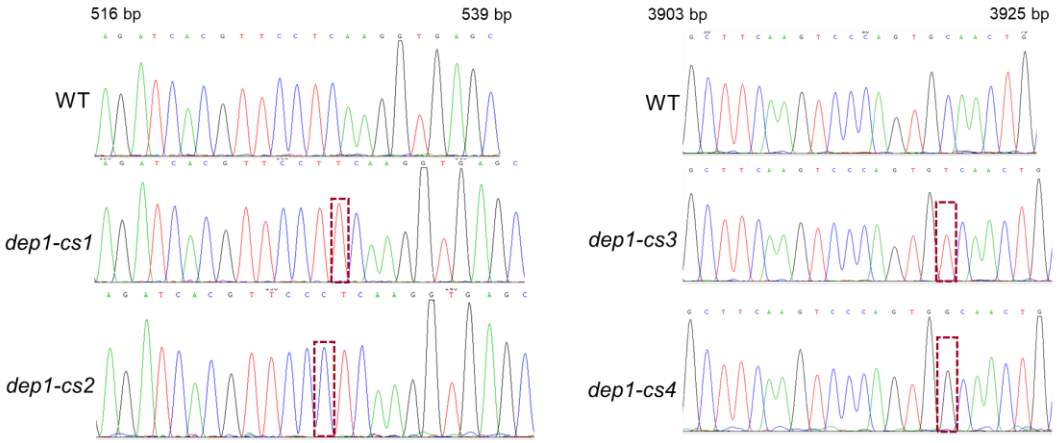

Figure S1. Sequencing confirmation of the *DEP1* gene in WT and *dep1-cs* transgenic plants generated by the CRISPR/Cas9 approach.

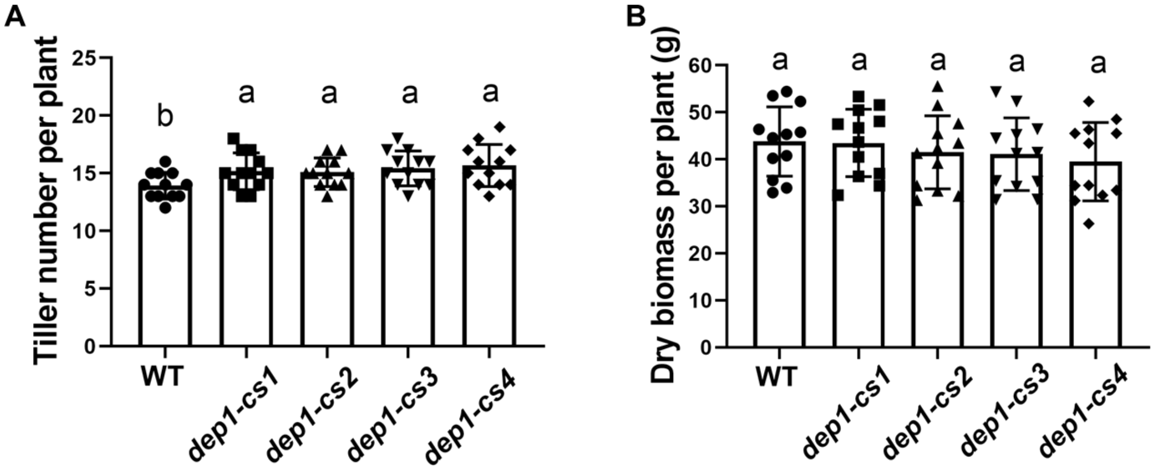

Figure S2. Agronomic traits of wild-type (WT) and *dep1-cs* plants. **A** Tiller number per plant. **B** Dry biomass per plant. Values are means, error bars are SD; n = 12 biological replicates; Significant differences are indicated by different letters ($P < 0.05$).

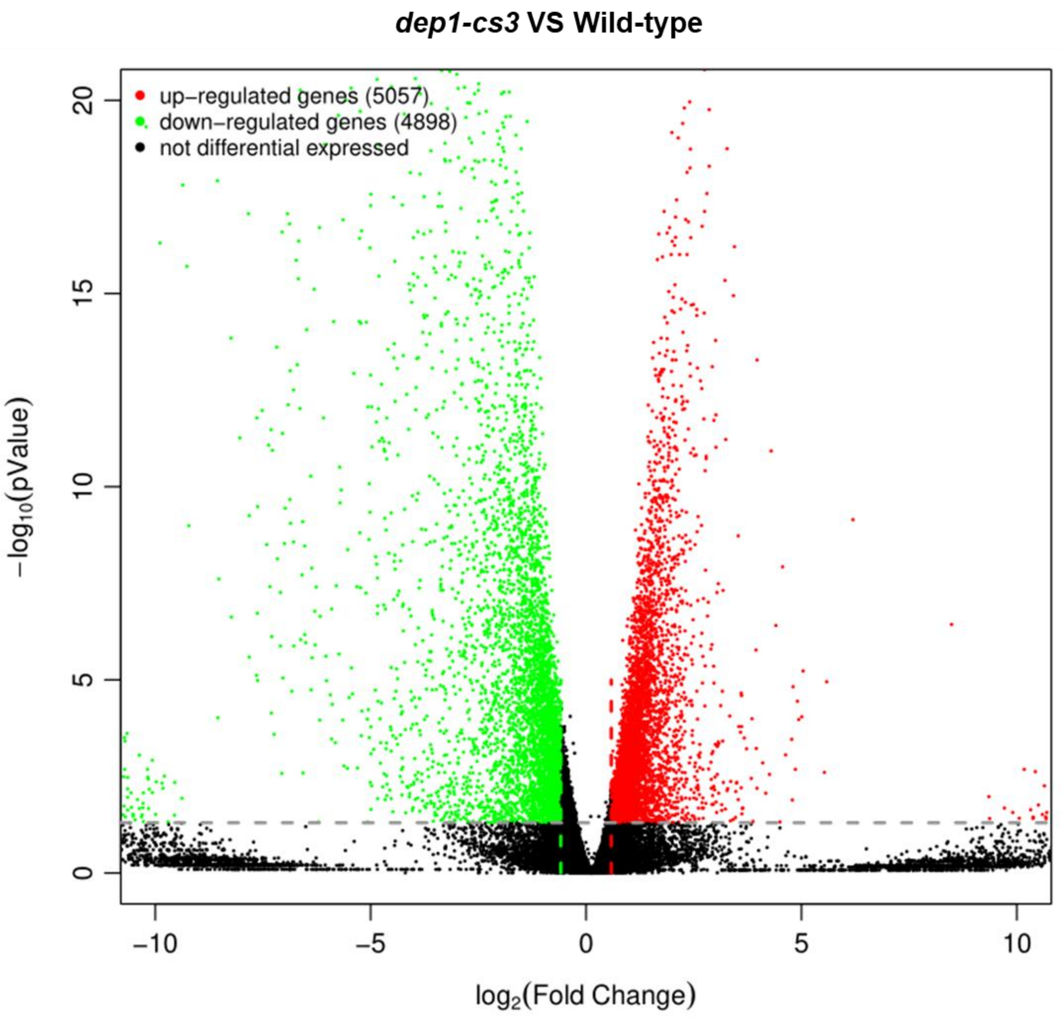

Figure S3. The visualization of differentially expressed genes (DEG) between wild-type and *dep1-cs* plants was performed by volcano plot, with the magnitude of the fold-change shown along the X-axis and the statistical significance (-log10 of *q*-value) shown on the Y-axis.

Table S1. Primers used in this study.

Gene	Locus	Forward primers	Reverse primers
ACT1	LOC_Os03g50885	ACCATTGGTGCTGAGCGTTT	CGCAGCTTCCATTCCTATGAA
CESA4	LOC_Os01g54620	AGGCAAGCGCTCTATGGTTA	GCATCGAGCGTTCATACTCA
CESA7	LOC_Os10g32980	TACGTCAACAACAGCAAGGC	TCCAGCCCCTTCATGTTGAT
CESA9	LOC_Os09g25490	TACAAGAACGGCAACCTCA	AAGAACAAACTCGCAAACG
IRX10	LOC_Os01g70200	GTGCCTGAACCACATGTTTG	AGAAATGATCAGCCCCCTCT
IRX9	LOC_Os07g49370	ACAAGGAGATGATGATTTTAGCA	AGGCGGAGGTCGTAGATGTCG
IRX14	LOC_Os06g47340	GATGGATGGTGTGATTGTG	TTGAACTGGAAGAGGCATA
XAT2	LOC_Os02g22480	GGCGAAGAGGATAATAAGAAC	TCACGGATGGTATAATTGGA
XAT3	LOC_Os03g37010	AAGCAAGAAGGTATGAATAATGT	AGTGGCAGTTGGATGAAT
PAL6	LOC_Os04g43800	TTTCTACAACAACGGCCTGC	TTGACGTCTTGGTTGTGCTG
PAL8	LOC_Os11g48110	AAGGCCGCTGTTAACAAGTG	GGATGTCACCGTTATTGGCC
4CL3	LOC_Os02g08100	AGGATGATCTTGCCGGTGAA	TGGCTCTCAAGTCCTTCCTG
CCR10	LOC_Os02g56700	GGCGATGATGAGAAGAAGCA	ACAGTGTCAGCCTCTCCTTG
CAD2	LOC_Os02g09490	CGACTCGCTGGACTACATCA	AGAAGTTGAGCACCTCCTCC
CAD7	LOC_Os04g52280	CATCATCAACACGGCCTCTG	AGTCGATCATCTCCTGCGTT
CAld5H1	LOC_Os10g36848	TCCAGGAGTTCTCCAAGCTG	CCATGTGCTCGTCGATGATC
